# Optimization of Weighting Window Functions for SAR Imaging via QCQP Approach

**DOI:** 10.3390/s20020419

**Published:** 2020-01-11

**Authors:** Jin Liu, Wei Wang, Hongjun Song

**Affiliations:** 1 Department of Space Microwave Remote Sensing System, Institute of Electronics, Chinese Academy of Sciences, Beijing 100190, China; ww_nudt@sina.com (W.W.); hjsong@mail.ie.ac.cn (H.S.); 2 Aerospace Information Research Institute, Chinese Academy of Sciences, Beijing 100094, China; 3 School of Electronic, Electrical and Communication Engineering, University of Chinese Academy of Sciences, Beijing 100039, China

**Keywords:** window function, convex optimization, peak sidelobe ratio, signal-to-noise ratio (SNR) loss, synthetic aperture radar (SAR) imaging

## Abstract

Weighting window functions are commonly used in Synthetic Aperture Radar (SAR) imaging to suppress the high Peak SideLobe Ratio (PSLR) at the price of probable Signal-to-Noise Ratio (SNR) loss and mainlobe widening. In this paper, based on the method of designing a mismatched filter, we have proposed a Quadratically Constrained Quadratic Program (QCQP) approach, which is a convex that can be solved efficiently, to optimize the weighting window function with both amplitude and phase, expecting to offer better imaging performance, especially on PSLR, SNR loss, and mainlobe width. According to this approach and its modified form, we are able to design window functions to optimize the PSLR or the SNR loss under different kinds of flexible and practical constraints. Compared to the ordinary real-valued and symmetric window functions, like the Taylor window, the designed window functions are complex-valued and can be asymmetric. By using Synthetic Aperture Radar (SAR) point target imaging simulation, we show that the optimized weighting window function can clearly show the weak target hidden in the sidelobes of the strong target.

## 1. Introduction

In many high-power radar systems like the Synthetic Aperture Radar (SAR) system, the Linear Frequency Modulation (LFM) signal is used, which is also known as the chirp signal [1]. The LFM signal can achieve a high resolution by the pulse compression technique, which is usually realized by applying a matched filter [2]. However, the Peak SideLobe Ratio (PSLR) of the matched filter output is about −13dB. This can be a problem in a multiple-target case with highly varied strong and weak scatters, indicating that the weak targets may be buried in the sidelobes of the strong scatters, which thus may reduce imaging quality and affect possible further processing, like target detection and recognition.

In order to mitigate this high PSLR problem, a commonly used solution is to apply window functions with different trade-offs at the reception side [3,4,5,6,7]. However, applying window functions to the matched filter at the reception side may cause Signal-to-Noise Ratio (SNR) loss, and may also broaden the mainlobe. Besides, the existing window functions may not be able to offer the performance with Pareto optimality [8] if we take PSLR, SNR loss as objectives and mainlobe width (which can be calculated from the mainlobe shape) as a constraint. Therefore, it is meaningful to design the window function in an optimization sense.

A matched filter followed by a window function actually forms a mismatched filter. Therefore, a new window function can be obtained by a mismatched filter as long as we know the specific information about the matched filter. There are many previous works on mismatched filter designs, which can be referred to the design window function. In [9], a mismatched filter was designed for burst waveforms by using nonlinear programming techniques. A least square method was proposed in [10] to design the mismatched filter for the phase code. Later, the linear programming techniques were used for a mismatched filter design [11]. This method can solve the PSLR problem, but it is not applicable to complex values. Iterative methods have been proposed to design mismatched filters in [12] to minimize the PSLR, and later in [13]. A minimum mean-square error estimation method was used to suppress range sidelobes for phase codes [14]. In [15], to solve the PSLR problem directly, an $L_{p}$-norm was replaced by an $L_{2p}$-norm with p>1. The authors used a matched filter followed by a parameterized multiplicative finite impulse response filter to form a mismatched filter, to suppress the sidelobe of the Barker code [16]. In [17], two or more mismatched filters were used to suppress the sidelobe. A convex optimization method was proposed to optimize the PSLR and the Peak Cross Correlation Level (PCCL) of the transmitted phase code of 40 points in a Multi-Input Multi-Output (MIMO) radar system [18]. Furthermore, in [19], the authors let *p* in [15] be sufficiently large to approximately solve the PSLR problem. Later in [20,21], a Quadratically Constrained Quadratic Program (QCQP) method was proposed to minimize the PSLR. In [7], the Integrated SideLobe Ratio (ISLR) of the mismatched filter response of Kasami code was optimized. In [1], a least square mismatched filtering approach was proposed to minimize sidelobe levels for frequency modulation waveforms. A convex optimization method with semidefinite relaxation was proposed in [22,23]. In [22], the authors took the maximum SNR loss as a constraint to optimize the PSLR and the PCCL, while in [23], the authors took the PSLR and the PCCL as constraints to optimize the interference and jamming power. In recent years, a simultaneous optimization of the waveform and mismatched filter was proposed in [24] to suppress the sidelobe. In [25], the authors used the convex optimization method to design the ultra-low sidelobe for the LFM signal. In [26], a new performance metric of the sidelobe was proposed, which was then to be optimized with the design of the mismatched filter by using the closed-form solution and the iterative method. All these previous works used different methods to design mismatched filters to deal with various kinds of optimization objects and constraints. However, most of the mismatched filters focus on code waveforms, like the phase code, which may suffer much larger distortion in a high-power, wide bandwidth radar system than the LFM signal may [1,27]. Besides, most of the optimizations of mismatched filters have not taken enough constraints into account, such as a clearly defined mainlobe width, which will affect the resolution in SAR imaging. Thus, specific constraints on mainlobe width are needed in the design of a mismatched filter. Moreover, most of the mismatched filters are designed to optimize the PSLR or ISLR, but sometimes, an optimized SNR loss is preferred because the SNR loss is also a key factor in the quality of the SAR image. Finally, some approaches may not be able to provide imaging performance with Pareto optimality. Therefore, to design the window function with better performance for SAR imaging, we shall take these issues into account.

In this paper, we reformulate the PSLR optimization problem of the window function as an equivalent QCQP, and this QCQP is convex, thus meaning it can be solved effectively and efficiently by convex optimization methods, such as interior point methods [28,29]. Based on this framework, we design a window function by using the convex optimization method. Then, we compare the results with the Taylor window function output, showing that this approach is an effective, efficient, and flexible optimization method that can provide better imaging performance and accommodate diverse cases, like requirements on PSLR or SNR loss. This paper is organized as follows: in Section 2, we introduce the optimization model and method. In Section 3, we give the window function design examples based on the optimization method described in Section 2. In Section 4, the SAR point target imaging simulation is shown. Conclusions are drawn in Section 5.

## 2. Optimization Model for Window Function

In many active radar imaging systems like the SAR system, we transmit a signal to the interested region and process the received echo to get the imaging results. Let $s={\left(s_{1},s_{2},\cdots ,s_{N}\right)}^{⊤}$ be the sampled transmitted signal vector. The output at the reception side after being processed by a filter $q_{K\times 1}$ with its length K=N+2p≥N with p≥0, can be written as:

(1) $$y_{(K+N-1)\times 1}=\Lambda_{K}\left(s\right)\times q_{K\times 1}.\;$$

The matrix $\Lambda_{K}\left(s\right)$ is a Toeplitz matrix with dimension (K+N−1)×K formed by the signal vector s, as below:

(2) $$\Lambda_{K}\left(s\right)={\left[\begin{array}{ccccccc} s_{N} & 0 & \cdots & \cdots & \cdots & \cdots & 0\\ \vdots & s_{N} & \ddots & & & & \vdots \\ s_{2} & \vdots & \ddots & 0 & & & \vdots \\ s_{1} & s_{2} & \cdots & s_{N} & 0 & \cdots & 0\\ 0 & s_{1} & \ddots & \vdots & s_{N} & \ddots & \vdots \\ \vdots & \ddots & \ddots & s_{2} & \vdots & \ddots & 0\\ 0 & \cdots & 0 & s_{1} & s_{2} & \cdots & s_{N}\\ \vdots & & & 0 & s_{1} & \ddots & \vdots \\ \vdots & & & & \ddots & \ddots & s_{2}\\ 0 & \cdots & \cdots & \cdots & \cdots & 0 & s_{1} \end{array}\right]}_{(K+N-1)\times K}.\;$$

If $q=s^{*}$, which implies K=N, it is the matched filter case and its output can be written as $y_{\mathrm{MF}}=\Lambda_{N}\left(s\right)\cdot s^{*}$. Here, $s^{*}={\left(s_{1}^{*},s_{2}^{*},\cdots ,s_{N}^{*}\right)}^{⊤}$, and the ${\left(\cdot \right)}^{*}$ is the conjugate operator. Usually, a suitable window function is then chosen to suppress the high sidelobe of the output to an acceptable level. Rather than applying a commonly used window function, we here combine the matched filter and the window function in a so-called mismatched filter to directly optimize at the reception side, expecting to get better imaging performance. If we set K=N for the mismatched filter, then we can further obtain the optimized window function by excluding the matched filter based on the optimized mismatched filter. Thus, the object here is to find the mismatched filter sequence q which minimizes the PSLR of the filter output, that is:

(3) $$\begin{array}{cc} \min_{q} & {\;∥Fy∥}_{\infty }\\ s.t. & \;s^{H}q=s^{H}s. \end{array}\;$$

This is a minimax optimization problem. ${∥x∥}_{\infty }$ denotes the infinity norm of vector x, that is, $\max_{j}\left|x_{j}\right|$. F is a diagonal matrix with 1 or 0 on the diagonal to screen out the points belonging to the sidelobe of the filter output y obtained in (1). For example, if we define the only point in the middle of y as the mainlobe, which is usually applied in the phase code, then F=diag(1,⋯,1,0,1,⋯,1) with a 0 at the middle of the diagonal. However, for other signals like the LFM signal, which is widely used in SAR imaging, the mainlobe is usually wider (in points) than the phase code and we must have a different F to obtain the sidelobe of the LFM signal. The constraint here in (3) mainly aims at precluding the null solution q=0. To minimize the PSLR, it is equivalent to setting the constraint that all sidelobe points are no larger than a specific variable, then minimizing this variable. We actually found out that this minimax optimization problem (3) can be reformulated as the following:

(4) $$\begin{array}{c} \min_{q,a}\;\;a\\ s.t.\;s^{H}q=s^{H}s\\ \;{\left(\lambda_{N+p+i}\left(s\right)q\right)}^{H}\times \left(\lambda_{N+p+i}\left(s\right)q\right)\leq a,\;\left|i\right|>N_{\mathrm{ML}}. \end{array}\;$$

Here, *a* denotes the magnitude of the peak sidelobe level to be optimized, and $\lambda_{N+p+i}\left(s\right)$ is the (N+p+i)th row of the matrix $\Lambda_{K}\left(s\right)$. $N_{\mathrm{ML}}$ here denotes half of the mainlobe width (in points, without the middle point) of the filter output, and $N_{\mathrm{ML}}$ is in accordance with matrix F in defining the mainlobe.

Next, we add more constraints to (4). The SNR loss is related to the ratio between the SNR of the mismatched filter (i.e., matched filter with window function) and the SNR of the matched filter in the same signal and noise conditions. Without the SNR loss constraint, we cannot preclude the possibility that the expected low PSLR is obtained by a large SNR loss. The echo received can be written as x=s+v, where s is the signal and v denotes the noise, and we define the autocorrelation matrix of the noise as $R_{v}=E\left[{\mathrm{vv}}^{H}\right]$. After being processed by the filter h at the reception side, the echo has an output $y=h^{H}x=h^{H}s+h^{H}v=y_{s}+y_{v}$. Here, $h^{H}$ denotes the conjugate transpose of the matrix h, and $y_{s}$ and $y_{v}$ denote the output of the signal and the noise, respectively. By [2], we have the SNR:

(5) $$\begin{array}{c} \mathrm{SNR}=\frac{|y_{s}{|}^{2}}{E\{|y_{v}{|}^{2}\}}=\frac{|y_{s}{|}^{2}}{E\{\left(h^{H}v\right){\left(h^{H}v\right)}^{H}\}}=\frac{|h^{H}{s|}^{2}}{h^{H}R_{v}h}. \end{array}\;$$

We know from [2] that in the matched filter case, we have $h=\gamma R_{v}^{-1}s$. Assume the noise here is white noise, that is, $R_{v}=E\left[{\mathrm{vv}}^{H}\right]=\sigma^{2}I$. We have $h=\gamma /\sigma^{2}\cdot s$, and we can further get the SNR loss below. Note that the inequality in (6) can be obtained by the Cauchy–Schwarz inequality.

(6) $$\begin{array}{cc} \mathrm{SNR}\;\mathrm{Loss} & =10\log_{10}\left(\frac{{\mathrm{SNR}}_{\mathrm{mismatched}}}{{\mathrm{SNR}}_{\mathrm{matched}}}\right)\\ & =10\log_{10}\left(\frac{|q^{H}{s|}^{2}}{q^{H}R_{v}q}\times \frac{h^{H}R_{v}h}{|h^{H}{s|}^{2}}\right)\\ & =10\log_{10}\left(\frac{|q^{H}{s|}^{2}}{\left(q^{H}q\right)\left(s^{H}s\right)}\right)\leq 0. \end{array}\;$$

By applying the constraint in (3) to (6), we get

(7) $$\mathrm{SNR}\;\mathrm{Loss}=10\log_{10}\left(\frac{s^{H}s}{q^{H}q}\right).\;$$

We can use β(β≤0) as the SNR loss constraint, then we have SNRLoss≥β here. The constraint in (3) shows that q will be a nonzero vector, then (7) is equivalent to $q^{H}q\leq \alpha \times s^{H}s$, here $\alpha \;=\;10^{-\beta /10}\;\geq \;1$. We may also add the mainlobe constraint to the optimization problem. We can use $b={\left(b_{-N_{\mathrm{ML}}},b_{-N_{\mathrm{ML}}+1}\cdots ,b_{-1},b_{0},b_{1},\cdots ,b_{N_{\mathrm{ML}}-1},b_{N_{\mathrm{ML}}}\right)}^{⊤}$ as the mainlobe constraint. This vector b here actually gives the constraints on the mainlobe shape, from which we can further calculate the mainlobe width. Thus, by (4), the mismatched filter optimization problem aiming at minimizing the PSLR of the receive filter output under the constraints can be written as follows:

(8) $$\begin{array}{c} \min_{q,a}\;\;a\\ s.t.\;s^{H}q=s^{H}s\\ \;\;q^{H}q\leq \alpha \times s^{H}s\\ \;{\left(\lambda_{N+p+i}\left(s\right)q\right)}^{H}\times \left(\lambda_{N+p+i}\left(s\right)q\right)\leq a,\;\left|i\right|>N_{\mathrm{ML}}\\ \;{\left(\lambda_{N+p+i}\left(s\right)q\right)}^{H}\times \left(\lambda_{N+p+i}\left(s\right)q\right)\leq b_{i},\;\left|i\right|\leq N_{\mathrm{ML}}. \end{array}\;$$

The constraints here are the non-null-solution constraint, the SNR loss constraint, the output constraints on the sidelobe, and the output constraints on the mainlobe, respectively. The parameters of the latter three constraints can be obtained by the output of applying a specific window function, or just simply using some proper values as constraints. By solving this convex optimization problem (8), we can get the optimized window function from the sequence q and the value of *a*, which can further give the PSLR.

Furthermore, we can also optimize the SNR loss under the constraints of PSLR and mainlobe width. With a mathematical modification of (8), we can obtain the optimization problem (9), which aims at minimizing the SNR loss:

(9) $$\begin{array}{c} \min_{q}\;{∥q∥}_{2}\\ s.t.\;s^{H}q=s^{H}s\\ \;{\left(\lambda_{N+p+i}\left(s\right)q\right)}^{H}\times \left(\lambda_{N+p+i}\left(s\right)q\right)\leq a_{0},\;\left|i\right|>N_{\mathrm{ML}}\\ \;{\left(\lambda_{N+p+i}\left(s\right)q\right)}^{H}\times \left(\lambda_{N+p+i}\left(s\right)q\right)\leq b_{i},\;\left|i\right|\leq N_{\mathrm{ML}}. \end{array}\;$$

From (3), (6), and (7), we know that $s^{H}s\leq q^{H}q$, and in order to minimize SNR loss here, we can equivalently minimize ${∥q∥}_{2}$. $a_{0}$ is a predefined constant which gives the PSLR constraint. The remaining two constraints in (9) are just the same as the constraints in (8). This optimization problem in (9) is also a QCQP optimization problem, which can be solved efficiently. By solving the optimization problem (9), we can obtain the window function that optimizes SNR loss.

Moreover, in some specific applications, a special shape of the sidelobe may be desired, and we may add the new constraints on the sidelobe shape to obtain a new convex optimization problem as long as the new constraints remain convex. For example, we can add sidelobe shape constraints on the PSLR optimization problem (8). Here, we placed a gap as the special sidelobe shape near the mainlobe. We used $N_{\mathrm{nSL}}$ to denote the points in the near sidelobe, and the optimization problem is shown in (10) below:

(10) $$\begin{array}{c} \min_{q,a}\;\;a\\ s.t.\;s^{H}q=s^{H}s\\ \;\;\;q^{H}q\leq \alpha \times s^{H}s\\ \;{\left(\lambda_{N+p+i}\left(s\right)q\right)}^{H}\times \left(\lambda_{N+p+i}\left(s\right)q\right)\leq a,\;\left|i\right|>N_{\mathrm{nSL}}\\ \;\;{\left(\lambda_{N+p+i}\left(s\right)q\right)}^{H}\times \left(\lambda_{N+p+i}\left(s\right)q\right)\leq c,\;N_{ML}\leq \left|i\right|\leq N_{\mathrm{nSL}}\\ \;{\left(\lambda_{N+p+i}\left(s\right)q\right)}^{H}\times \left(\lambda_{N+p+i}\left(s\right)q\right)\leq b_{i},\;\left|i\right|<N_{\mathrm{ML}}. \end{array}\;$$

Here, we used *c* in (10) to denote the level of the near sidelobe. By solving the optimization problem (10), we could further get the optimized window function, which could give the special sidelobe shape. We could also take SNR loss as the optimization objective function like (9) does, and add other convex sidelobe shape constraints to meet the sidelobe requirements.

## 3. Optimized Window Function Design

In this section, we give the window function design examples by the method introduced in Section 2 with optimization results. We used the output of the well-performed and widely used Taylor window function as a comparison. Firstly, we give the optimization result of PSLR under the constraints of SNR loss and mainlobe width. Secondly, we optimize the SNR loss under the constraints of PSLR and mainlobe width. Thirdly, we show some sidelobe shape examples obtained by optimized window functions.

### 3.1. Window Function Design with Optimized PSLR

**Figure 1:**
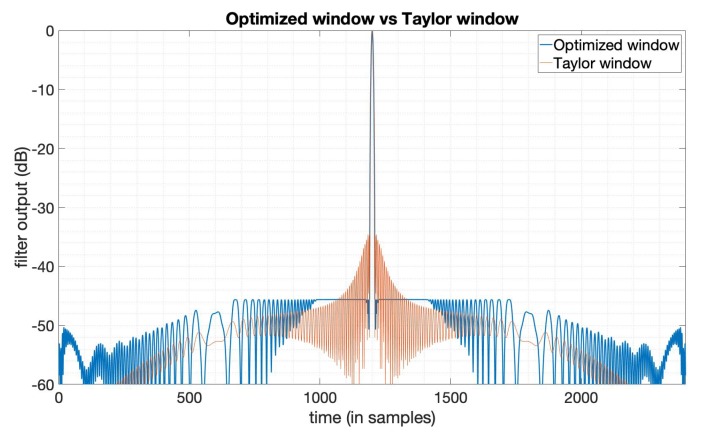
The output of the Taylor window ($\bar{n}=6,\mathrm{PSLR}=-35\;\mathrm{dB}$) and the PSLR -optimized window.

**Figure 2:**
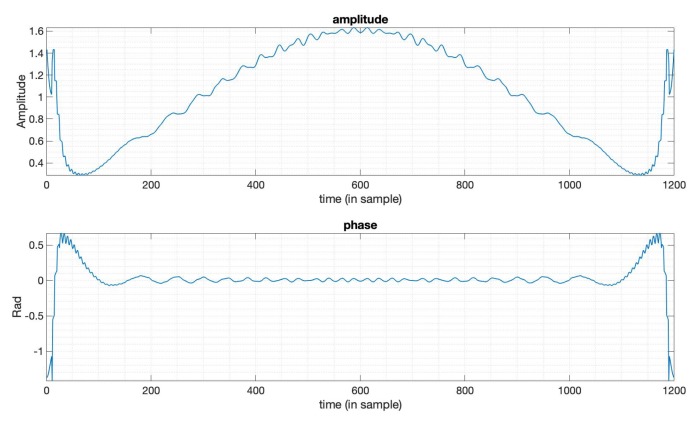
The amplitude and phase of the optimized window function in Figure 1.

The typical method used to suppress PSLR is achieved by applying window functions [6,7]. We used the Taylor window for comparison. The parameters used here of the Taylor window are $\bar{n}\;=\;6,\mathrm{PSLR}=-35\;\mathrm{dB}$. The $\bar{n}$ denotes the distance from the mainlobe for which the sidelobes are constant [5]. The LFM signal is used here as the transmitted signal with bandwidth 20MHz, duration 10 μs. Figure 1 gives the output results of the optimized window function (in blue line) and the Taylor window function (in orange line). We can see that the PSLR of the optimized window output is much lower than the output of the Taylor window. Figure 2 gives the amplitude function and phase function of the optimized window, which means if we apply this window just like the way we do with the Taylor window, to the LFM signal in this case, we can get the compression result shown in Figure 1. We can see that the optimized window in this case is a complex valued window, while window functions mentioned in [5] are all real-valued. Table 1 shows the specific values of the three key factors in SAR imaging, that is, PSLR, SNR loss, and mainlobe width. Note that the mainlobe width here is the width between the points on each side of the mainlobe where the power has fallen to −3dB of its peak. The values tell us that a lower PSLR is obtained with the constraints on SNR loss and mainlobe width being satisfied.

### 3.2. Window Function Design with Optimized SNR Loss

**Figure 3:**
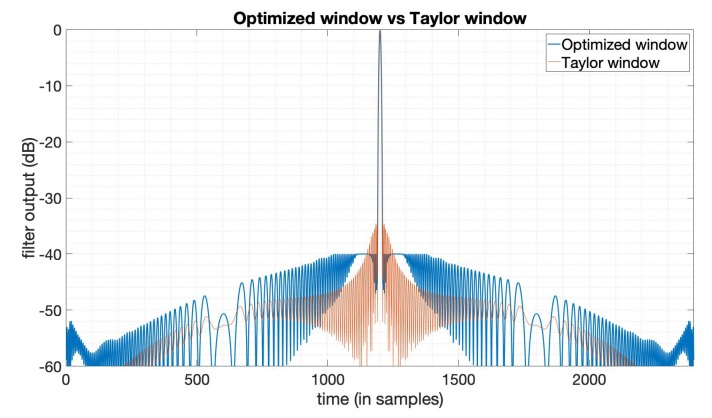
The output of the Taylor window ($\bar{n}=6,\mathrm{PSLR}=-35\;\mathrm{dB}$) and the SNR loss-optimized window.

**Figure 4:**
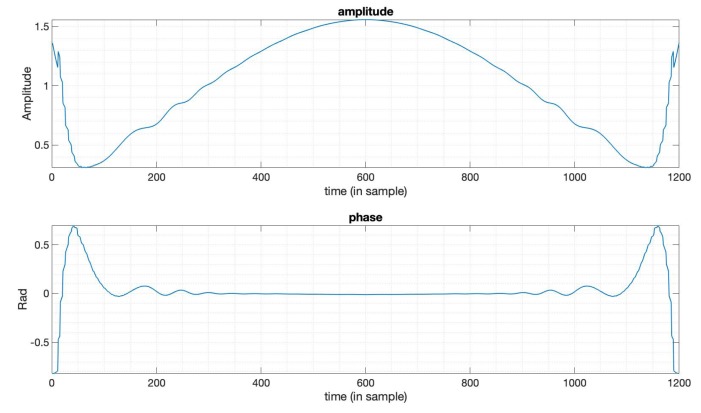
The amplitude and phase of the optimized window function in Figure 3.

In some cases, there is a predefined PSLR requirement in the SAR imaging task, and in the meantime, we also want an optimal SNR loss and an acceptable mainlobe width. In this subsection, we design a window function to optimize the SNR loss under the constraints on the PSLR and mainlobe width based on (9), to show the flexibility of this optimization method introduced in Section 2. Here, we still use the LFM signal with a bandwidth 20MHz, duration 10 μs. The Taylor window here used for comparison has the parameters $\bar{n}=6,\mathrm{PSLR}=-35\;\mathrm{dB}$. We set the PSLR constraint −40dB and use the mainlobe width of the output of the Taylor window here as mainlobe constraints. By solving the optimization problem (9), we obtain the optimization results. Figure 3 shows the output compared between the optimized window and Taylor window. Figure 4 is the amplitude function and phase function of the optimized window. We can see that the window in this case is still with an optimized amplitude and phase, while ordinary window functions are only real-valued. Table 2 gives the specific results from this optimization, showing that the SNR loss, PSLR, and mainlobe width in this case are all better than the Taylor window. The optimization results here not only show the flexibility of this method, but also indicate that we can set the constraints flexibly to a level with possible practical imaging requirements.

### 3.3. Optimized Window with Special Sidelobe Shape

In this subsection, we realize some of the specific shapes of the sidelobes, which is hard to obtain by simply applying ordinary window functions. Also, by this design method, the overall performance of the optimized window output with a specific sidelobe shape is still better than applying an ordinary window function. More importantly, we may be able to find out the hidden weak targets by trying various window functions obtained by our methods with different sidelobe shapes to the same echo at the reception side. For example, in a scene with a strong scatter and probably several neighboring weak targets, we may try various mismatched filters with lower sidelobe gaps at different places of the sidelobe to check through the whole sidelobe. This method may improve the possibility of finding out weak targets in the scene than those without a special sidelobe shape.

**Figure 5:**
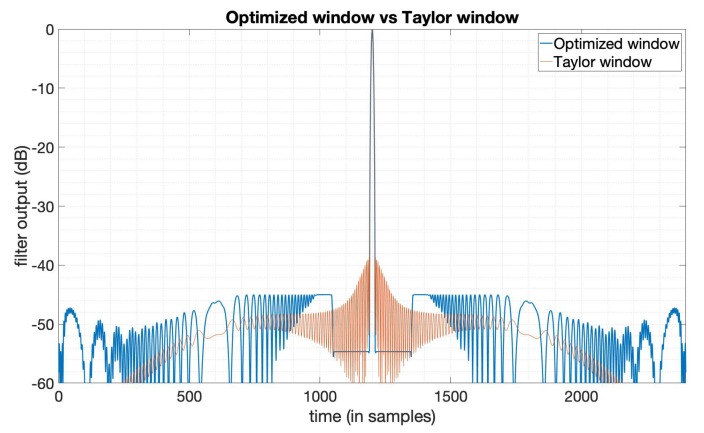
The output of the Taylor window ($\bar{n}=9,\mathrm{PSLR}=-40\;\mathrm{dB}$) and the PSLR-optimized window with symmetric sidelobe gaps.

**Figure 6:**
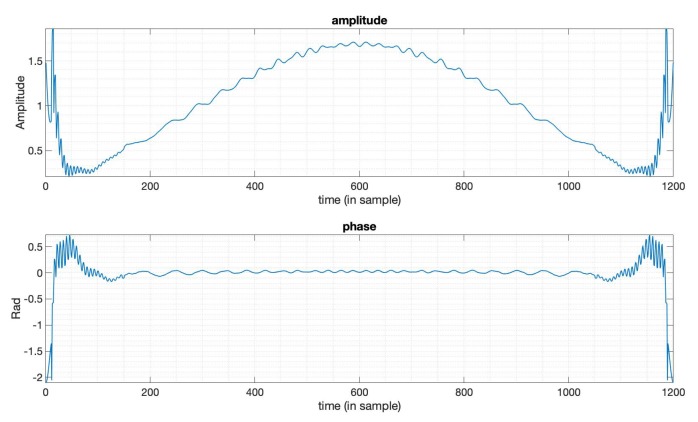
The amplitude and phase of the optimized window function in Figure 5.

Firstly, we can get a symmetric sidelobe gap near the mainlobe by solving the optimization problem (10). Results are shown in Figure 5. We can find out that there are two gaps at each side of the mainlobe. The sidelobe gaps can be optimized to a level of −54.65dB, and the constraints of the remaining sidelobe with a level of −45.00dB, the SNR loss and mainlobe width constraints generated by the Taylor window ($\bar{n}=9,\mathrm{PSLR}=-40\;\mathrm{dB}$) here are all satisfied. The optimized window function is shown in Figure 6, with both amplitude function and phase function.

**Figure 7:**
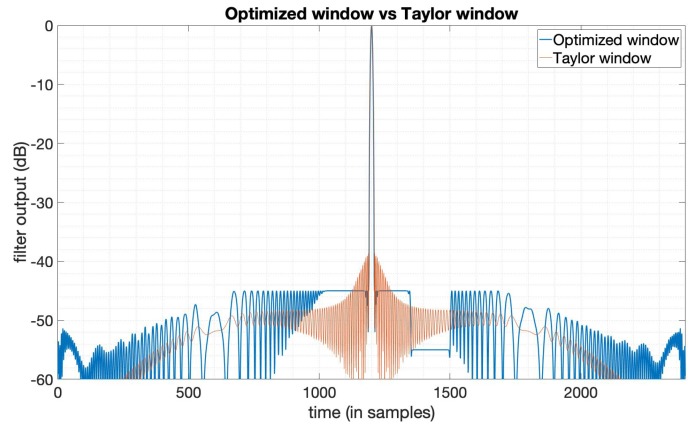
The output of the Taylor window ($\bar{n}=9,\mathrm{PSLR}=-40\;\mathrm{dB}$) and the SNR loss-optimized window with asymmetric sidelobe gap.

**Figure 8:**
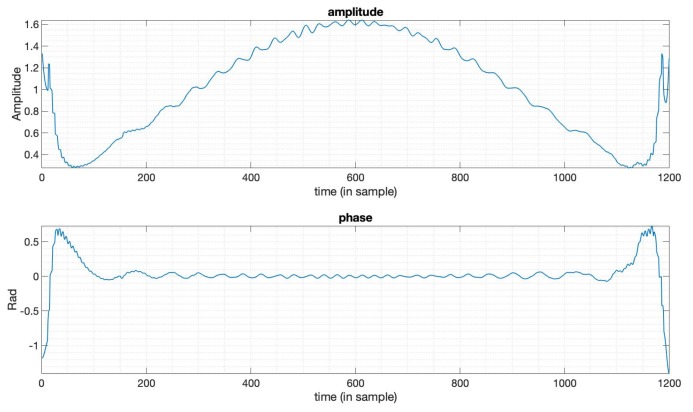
The amplitude and phase of the optimized window function in Figure 7.

Secondly, we can obtain an asymmetric sidelobe gap in the middle of the sidelobe by solving an optimization problem based on (9), aiming at optimizing the SNR loss. From Figure 7, an asymmetric sidelobe gap can be found on the right side of the mainlobe. The sidelobe gap is set to a level of −55dB and the remaining sidelobe is set to −45dB as constraints, and the mainlobe width constraints generated by the Taylor window ($\bar{n}=9,\mathrm{PSLR}=-40\;\mathrm{dB}$) are all satisfied. We can find from Figure 8 that in this case, the optimized window function is not symmetric anymore, both in amplitude function and phase function.

We used sidelobe gaps in this subsection as examples to show that by using this convex optimization method, we can flexibly set the place and the symmetry of the sidelobe gap, and we can also optimize either PSLR or SNR loss. These optimized windows can provide even better overall performance with special sidelobe shapes than the Taylor window function compared here. We can use the designed window functions with different kinds of sidelobe shapes to realize better detection performance by fusion processing on weak targets, which may probably be hidden by the sidelobes of strong scatters.

## 4. SAR Point Target Imaging Simulation

**Figure 9:**
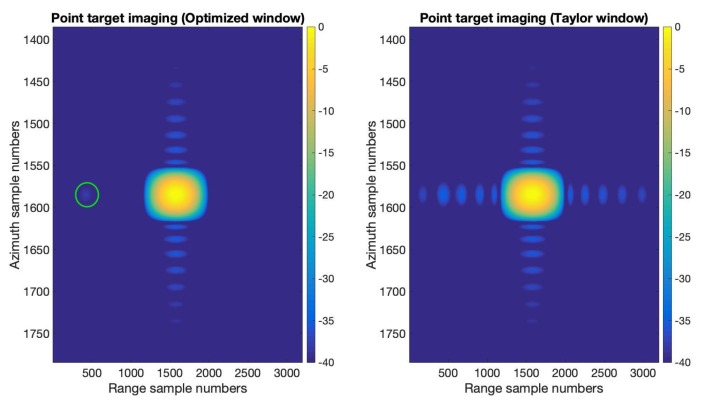
The images of the interpolated results (after upsampling) of the optimized window and the Taylor window ($\bar{n}=6,\mathrm{PSLR}=-35\;\mathrm{dB}$).

**Figure 10:**
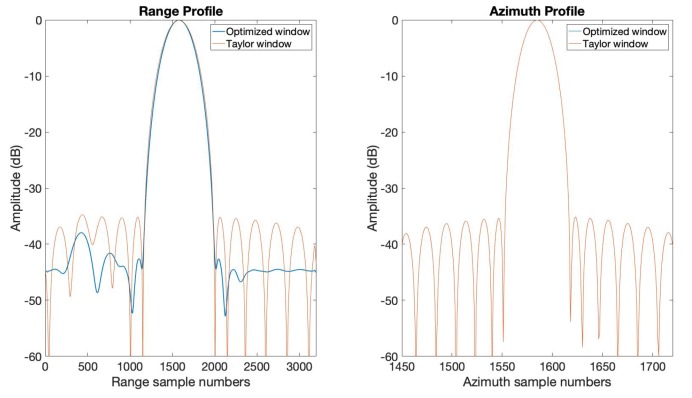
The range and azimuth profiles of the point targets in Figure 9.

We provide the results of applying the optimized window function obtained in the previous section to SAR imaging in this section. A two-targets scene with highly varied strength was set to verify the effectiveness of the optimized window function. A strong target was placed on the center of the scene, and a weak target was placed on the left side of the center with the same azimuth location. The Radar Cross-Section (RCS) difference of the two point targets was 40dB. The range-Doppler algorithm introduced in [30] was used for SAR imaging here. We realized this SAR point targets imaging simulation with two different sets of window functions. The first one is applying the optimized window function obtained in Section 3.1 in range and applying the Taylor window ($\bar{n}=6,\mathrm{PSLR}=-35\;\mathrm{dB}$) function in azimuth, and the second one is applying the Taylor window ($\bar{n}=6,\mathrm{PSLR}=-35\;\mathrm{dB}$) function both in range and azimuth. The remaining settings in this point target simulation are exactly the same. The imaging results are shown in Figure 9 and Figure 10. Figure 9 gives the interpolated plots of the point targets. In the optimized window case, we can see the weak target in the green circle shown in Figure 9, while in the Taylor window case, the weak target is buried in the sidelobe of the strong target. Figure 10 shows the range and azimuth profiles of the strong target. We can see in the range profile that the optimized window clearly displays a weak target on the left of the strong target. The azimuth profile of the optimized window and the Taylor window are the same. These SAR point target imaging simulation results have verified the effectiveness of the optimized window in this paper.

## 5. Conclusions

In this paper, we began with the PSLR optimization problem of the mismatched filter output. To optimize the PSLR of the weighting window, we reformulated the original optimization problem to a convex optimization problem. Based on this method, we were able to flexibly optimize the PSLR or SNR loss with proper constraints. Window function design examples with different optimization objectives have been shown to verify the effectiveness of this method. By extending the convex optimization problem, we developed the sidelobe shape technique, which can be realized by an optimized window function, and this can meet some special sidelobe shape requirements or possibly find out the weak targets by applying different kinds of sidelobe shapes. Finally, by SAR point target simulation, we have verified the effectiveness of the optimized weighting window function.

## Figures and Tables

**Table 1:** Comparison between output parameters of Taylor window ($\bar{n}=6,\mathrm{PSLR}=-35\;\mathrm{dB}$) and PSLR-optimized window

	Taylor Window	Optimized Window
PSLR	−34.60 dB	−45.62 dB
SNR Loss	−0.92 dB	−0.92 dB
Mainlobe width	1	95.18%

**Table 2:** Comparison between output parameters of Taylor window ($\bar{n}=6,\mathrm{PSLR}=-35\;\mathrm{dB}$) and SNR loss-optimized window

	Taylor Window	Optimized Window
SNR Loss	−0.92 dB	−0.78 dB
PSLR	−34.60 dB	−40.00 dB
Mainlobe width	1	92.77%
